# Depletion of thymopoietin inhibits proliferation and induces cell cycle arrest/apoptosis in glioblastoma cells

**DOI:** 10.1186/s12957-016-1018-y

**Published:** 2016-10-19

**Authors:** Lin Zhang, Gan Wang, Shiwen Chen, Jun Ding, Shiming Ju, Heli Cao, Hengli Tian

**Affiliations:** Department of Neurosurgery, Shanghai Jiao Tong University Affiliated Sixth People’s Hospital, School of Medicine, Shanghai Jiao Tong University, No. 600 Yishan Road, Xuhui District, Shanghai, 200233 China

**Keywords:** TMPO, Glioblastoma, Cell proliferation, Apoptosis

## Abstract

**Background:**

Glioblastoma (GBM) is the most malignant nervous system tumor with an almost 100 % recurrence rate. Thymopoietin (TMPO) has been demonstrated to be upregulated in various tumors, including lung cancer, breast cancer, and so on, but its role in GBM has not been reported. This study was aimed to determine the role of TMPO in GBM.

**Methods:**

Publicly available Oncomine dataset analysis was used to explore the expression level of TMPO in GBM specimens. Then the expression of TMPO was knocked down in GBM cells using lentiviral system, and the knockdown efficacy was further validated by real-time quantitative PCR and western blot analysis. Furthermore, the effects of TMPO silencing on GBM cell proliferation and apoptosis were examined by MTT, colony formation, and flow cytometry analysis. Meanwhile, the expression of apoptotic markers caspase-3 and poly(ADP-ribose) polymerase (PARP) were investigated by western blot analysis.

**Results:**

This study observed that the expression of TMPO in GBM specimens was remarkably higher than that in normal brain specimens. Moreover, knockdown of TMPO could significantly inhibit cell proliferation and arrest cell cycle progression at the G2/M phase. It also found that TMPO knockdown promoted cell apoptosis by upregulation of the cleavage of caspase-3 and PARP protein levels which are the markers of apoptosis.

**Conclusions:**

The results suggested TMPO might be a novel therapeutic target for GBM.

## Background

Glioblastoma (GBM) (World Health Organization grade IV astrocytoma) is the most frequent and most malignant brain tumor. The standard therapy method for newly diagnosed GBM is surgery followed by radiotherapy plus temozolomide chemotherapy [1]. But the GBM is still along with a 100 % recurrence rate and about a 5 % 5-year survival rate [2, 3]. Researchers are screening new drugs for GBM treatment, such as bevacizumab, which is a humanized monoclonal antibody against vascular endothelial growth factor A (VEGF-A), but it cannot improve overall survival in patients with GBM; the adverse events such as increased symptom severity are more serious [4, 5]. So the development of new therapeutic targets is essential for GBM therapy.

Thymopoietin (TMPO) is also named as lamina-associated polypeptide 2 (LAP2) and has six alternatively spliced isoforms with different functions [6]. TMPO can interact with lamins and BAF to regulate the organization of the nuclear structure and the dynamics of the cell cycle [7]. Recently, the role of TMPO in cancer biology has been reported [8]. LAP2β is upregulated in the rapidly proliferating cells of various hematological malignancies but is normally expressed in the slowly proliferating cells of chronic malignant hematological diseases [9]. LAP2β is upregulated in digestive tract tumor tissues and cells; its knockdown could inhibit migration, invasion, and metastasis but has no effect on cell proliferation. In pancreatic cancer cells, knockdown of LAP2β not only inhibits cell proliferation but also suppresses migration, invasion, and metastasis [10].

However, the role of TMPO in GBM has not been reported until now. In this study, short hairpin RNA (shRNA) for TMPO was used to knock down TMPO in GBM cells and the MTT assay and colony formation assay were used to determine the effect of TMPO on cell proliferation. Finally, the approach of TMPO that regulated cell proliferation, cell cycle progression, or apoptosis was determined. In addition, the knockdown of TMPO reduced the cell proliferation rate caused by promoting apoptosis.

## Methods

### Data mining and Oncomine analysis

A series of microarray datasets for glioblastoma were retrieved from the public Oncomine cancer microarray database (www.oncomine.org) to investigate TMPO expression in glioblastoma [11]. Differential expressions of TMPO between glioblastoma and normal tissues were retrieved from seven different databases, including French Brain [12], Liang Brain [13], Murat Brain [14], TCGA Brain (The Cancer Genome Atlas-Glioblastoma Multiforme Gene Expression Data, http://tcga-data.nci.nih.gov/tcga/), Shai Brain [15], Sun Brain [16], and Pomeroy Brain [17]. The TMPO gene expression in glioblastoma was compared with normal brain tissues according to the previously described [18].

### Cell lines and culture

GBM cells including U251 and U87 and human embryonic kidney cell line 293T were purchased from the Cell Bank of Chinese of Science (Shanghai, China). U251 was cultured in DMEM (SH30243.01B, HyClone, USA) supplemented with 10 % fetal bovine serum (FBS, S1810, Biowest, Spain). U87 was cultured in EMEM (SH30024.01B, HyClone, USA) supplemented with FBS, 1 % PEP, and 1 % NEAA. 293T cells which were used to package lentivirus were cultured in DMEM supplemented with 10 % FBS. All cells were maintained in a humidified atmosphere at 37 °C with 5 % CO_2_.

### Vector construction and virus infection

The sequences (S1: 5′-GCACAGATTCTTAGCTCAGAT-3′, S2: 5′-CTTGTGAAATACGGAGTGAAT-3′) were used as the target sequence to downregulate the TMPO (NM_003276.2) level, and shRNA oligos for TMPO were cloned into the pFH-L lentiviral vector (Hollybio, Shanghai, China) which carried green fluorescent protein (GFP) and were indicated as TMPO-shRNA and TMPO-shRNA(S2). The sequence (5′-TTCTCCGAACGTGTCACGT-3′) was used as a scramble control and cloned into the pFH-L vector (Con-shRNA). Then the shRNA plasmid and two pHelper plasmids pVSVG-I and pCMVΔR8.92 were co-transfected into 293T cells using the calcium phosphate transfection protocol. The viral supernatants were collected and filtered at 48 h after transfection. For lentivirus infection, U251 and U87 cells were seeded in six-well plates at a density of 7 × 10^4^ cells/well and 5 × 10^4^ cells/well, respectively. Subsequently, the lentiviruses containing TMPO-shRNA, TMPO-shRNA(S2), or Con-shRNA were transduced into U251 and U87 cells at 10 and 8 MOI (multiplicity of infection), respectively. After a 72-h infection, the infection efficiency was determined by observing the GFP expression under a fluorescence microscope.

### RNA extraction and real-time quantitative PCR

Total RNA was extracted using RNAiso Plus reagent (Takara, Japan) according to the manufacturer’s instructions and synthesized into complementary DNA (cDNA) using HiScript 1st Strand cDNA Synthesis Kit (Vazyme, China). TMPO expression was quantified using HiScript® Q RT SuperMix for qPCR (Vazyme, China) in a BioRad Connet Real-Time PCR platform and performed in triplicates. Actin was used as an endogenous control for normalization. The relative expression of TMPO was calculated as using the 2^−ΔΔCt^ method [19]. Ct was the threshold cycle of each transcript. The real-time quantitative PCR primers were shown as follows: TMPO: forward, 5′-TGCTCGCCTCCTGCCTGTAG-3′ and reverse, 5′-GACACAAAGCCAAGCCAGACC-3′; Actin: forward, 5′-GTGGACATCCGCAAAGAC-3′ and reverse, 5′-AAAGGGTGTAACGCAACTA-3′.

### Western blot

Total protein was extracted from cells using RIPA buffer (50 mM Tris-HCl, at pH 8.0, 150 mM NaCl, 5 mM EDTA, 0.1 % SDS, and 1 % NP-40). The protein samples (30 μg/well) were loaded and separated by 10 % SDS-polyacrylamide gel electrophoresis (PAGE). Western blot was performed according to the previous report [20]. Anti-TMPO (1:1000, 14651-1-AP, Proteintech, USA), poly(ADP-ribose) polymerase (PARP;1:1000, #9542, Cell Signaling Technology, USA), and caspase-3 (1:500, Cell Signaling Technology, USA) antibodies were used. An anti-GAPDH antibody (1:500000, 10494-1-AP, Proteintech, USA) was used as the loading control. The secondary antibody used was horseradish peroxidase (HRP)-conjugated goat anti-rabbit antibody (1:5000, SC-2054, Santa Cruz, USA).

### Cell proliferation analysis by MTT

After lentivirus infection for 72 h, a total density of 2500 cells/well for U251 cells or 3000 cells/well for U87 cells were reseeded in a 96-well plate. The cell viability was determined at five different timing points (days 1, 2, 3, 4, and 5) using the MTT assay. Briefly, 20 μl of MTT (Sigma, USA) solution was added to the wells and incubated for 4 h at 37 °C. Then 100 μl acidic isopropanol solution (10 % SDS, 5 % isopropanol, and 0.0 1 mol/l HCl) was added and incubated overnight to solubilize the formazan. The optical density (OD) value was measured at 570 nm using a microplate reader (Epoch, BioTek, USA). All experiments were performed in triplicates.

### Colony formation assay

Colony formation assay was carried out according to the previous report [21]. Briefly, U251 cells stably transduced lentivirus were reseeded on six-well plates and cultured for 12 days at a density of 600 cells/well. Then colonies were fixed using 4 % paraformaldehyde for 5 min and then stained with 1 % crystal violet for 30 s. The cells were photographed, and the number of colonies was counted.

### Flow cytometric analysis of the cell cycle

After lentivirus infection for 5 days, a total density of 80,000 cells/dish for U251 cells were reseeded in 6-cm dishes and continued to culture for 72 h. Then cells were collected, washed with pre-cooling PBS, and fixed with cooling 70 % ethanol overnight at 4 °C. After washing twice with pre-cooling PBS, the cells were resuspended and incubated in 500 μl PBS containing 50 μg/ml propidium iodide solution (PI) and 100 μg/ml RNase for 30 min at room temperature. Finally, the cells were analyzed on a FACS Calibur cytometer (BD, USA). Data were analyzed using CellQuest software.

### Apoptosis analysis

Apoptosis analysis was performed using the Annexin V-APC/7-AAD apoptosis kit (KeyGEN Biotech, Nanjing, China) according to the manufacturer’s instructions. Cells were analyzed on a FACS Calibur cytometer (BD, USA). Data were analyzed using CellQuest software.

### Statistical analysis

Statistical analysis was performed using SPSS 19 (SPSS Inc., USA). Results are shown as the mean ± standard deviation (STDEV) for at least three repeated individual experiments for each group. Statistical differences were determined using Student’s *t* test for independent samples. *p* < 0.05 was considered statistically significant.

## Results

### TMPO mRNA expression was elevated in glioblastoma

**Fig. 1:**
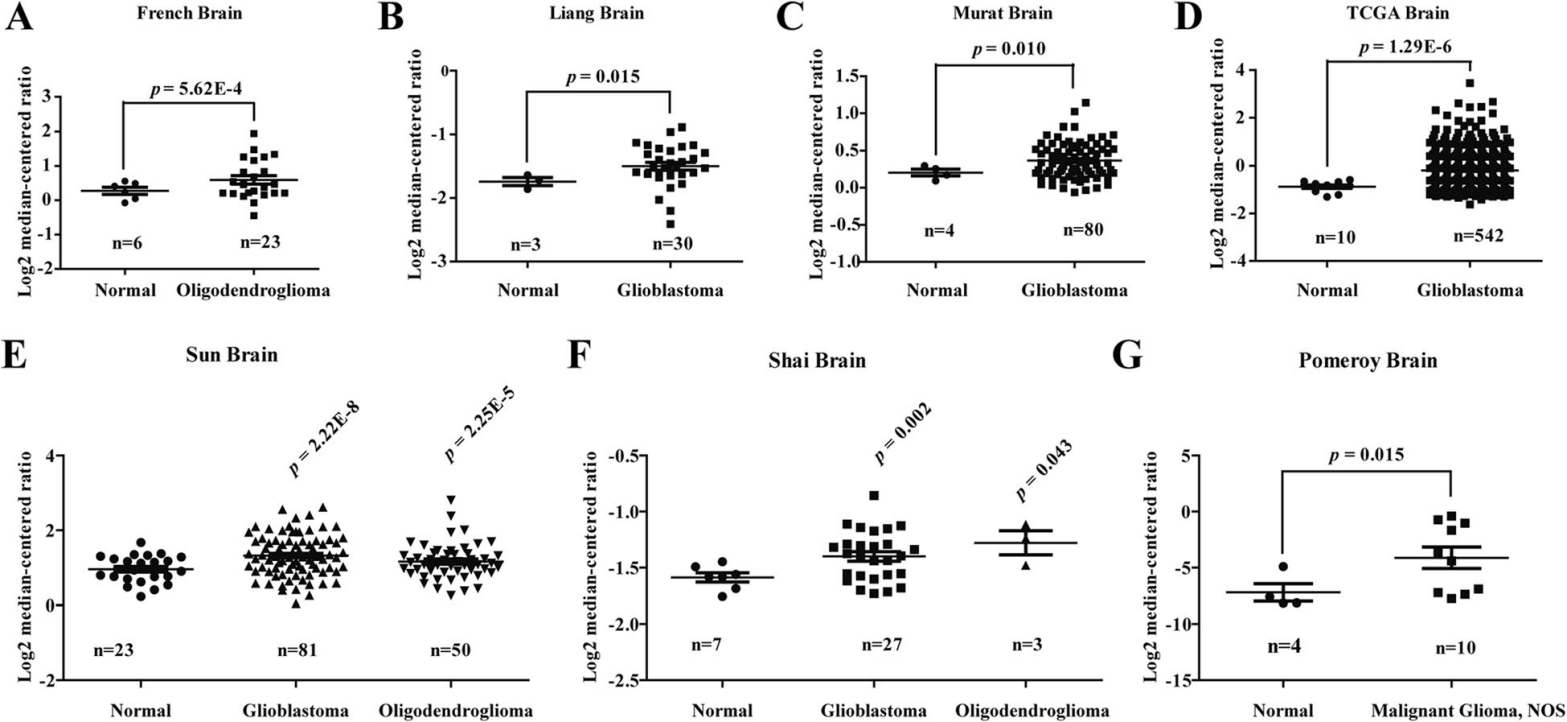
Upregulation of TMPO mRNA in GBM was revealed by Oncomine database. The expression of the TMPO gene was extracted and analyzed from **a** the French Brain dataset, **b** the Liang Brain dataset, **c** the Murat Brain dataset, **d** the TCGA Brain dataset, **e** the Shai Brain dataset, **f** the Sun Brain dataset, and **g** the Pomeroy Brain dataset shown as scatterplots

To analyze the expression level of TMPO in GBM, the publicly available Oncomine cancer microarray database was excavated. As shown in Fig. 1a, the French Brain dataset showed that TMPO expression is significantly elevated in oligodendroglioma (*n* = 23, *p* = 5.62E−4) compared with the normal tissues. Comparing with normal brain tissues, TMPO expression in glioblastoma tissues is much higher as shown in three different microarray datasets including the Liang Brain dataset (*n* = 30, *p* = 0.015), the Murat Brain dataset (*n* = 80, *p* = 0.010), and the TCGA Brain dataset (*n* = 542, *p* = 1.29E−6) (Fig. 1b–d). Also, TMPO expression was significantly increased in glioblastoma tissues (*n* = 27, *p* = 0.002) and oligodendroglioma tissues (*n* = 3, *p* = 0.043) in the Shai Brain dataset (Fig. 1e). Furthermore, the Sun Brain dataset revealed that TMPO was upregulated in glioblastoma tissues (*n* = 81, *p* = 2.22E−8) and oligodendroglioma tissues (*n* = 50, *p* = 2.25E−5) compared with normal brain tissues (Fig. 1f). The results of the Pomeroy Brain dataset were similar to the abovementioned results that the expression level of TMPO was also significantly higher in malignant glioma (*n* = 10, *p* = 0.015) than that in the normal tissues (Fig. 1g). These data suggested that TMPO might play an underlying carcinogenesis in glioblastoma.

### TMPO-shRNA could knock down TMPO effectively

**Fig. 2:**
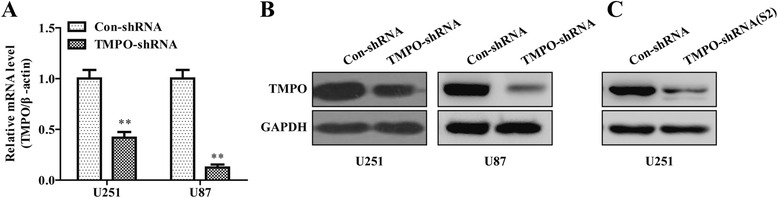
Knockdown efficiency of TMPO by lentivirus infection in the GBM cells. **a** Quantitative real-time PCR analysis of TMPO knockdown efficiency in U251 and U87 cells. The mRNA expression of TMPO was significantly suppressed when the cells were infected with TMPO-shRNA. **b** Western blot analysis validated TMPO-shRNA effectively downregulated the protein level of TMPO in U251 and U87 cells. **c** Western blot analysis confirmed TMPO-shRNA(S2) effectively decreased the protein expression of TMPO in U251 cells. **p < 0.01; *scale bars*, 10 μm

To determine the role of TMPO in GBM, TMPO-shRNA was used to knock down TMPO in U251 and U87. Quantitative real-time PCR revealed that the knockdown efficiency of TMPO in the messenger RNA (mRNA) level was about 60 % in U251 cells and up to 90 % in U87 cells (Fig. 2a). As shown in Fig. 2b, TMPO protein levels were reduced in both U251 and U87 cells infected with TMPO-shRNA by western blot assay. Moreover, western blot analysis also confirmed that the TMPO protein level in the TMPO-shRNA(S2) group was significantly decreased compared with that in the Con-shRNA group in U251 cells (Fig. 2c). These results suggested TMPO-shRNA constructed successfully significantly suppressed TMPO expression both at the mRNA and protein levels.

### Knockdown of TMPO inhibited cell proliferation

**Fig. 3:**
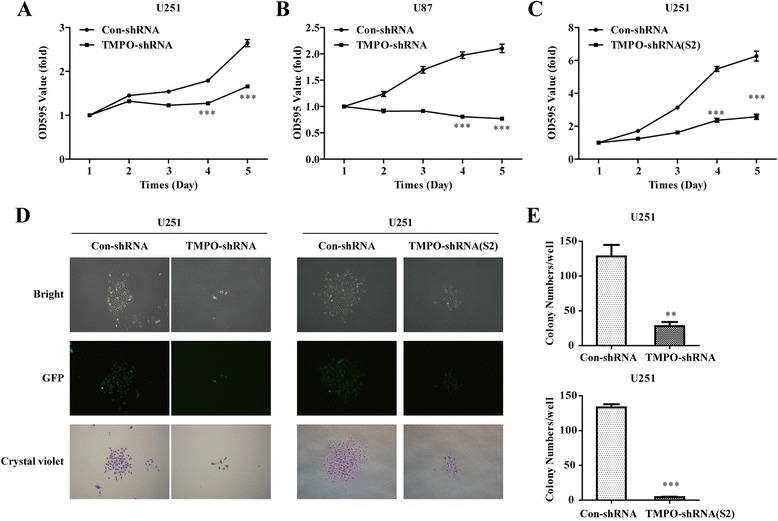
Downregulation of TMPO inhibited the proliferation of GBM cells. **a**, **b** Cell proliferation in U251 and U87 cells after TMPO-shRNA infection was determined by MTT assay, respectively. **c** The proliferation of U251 cells was examined after TMPO-shRNA(S2) infection by MTT assay. **d** Representative microscopic images of colonies in TMPO-shRNA- or TMPO-shRNA(S2)-infected U251 cells were stained by crystal violet. **e** Statistical analysis of the number of colonies. **p < 0.01, ***p < 0.001; *scale bars*, 25 μm

To determine whether TMPO affects GBM cell proliferative abilities, MTT and colony formation assays were carried out. As shown in Fig. 3a, b, the growth rate of the TMPO-shRNA group was significantly suppressed by 37.3 and 63.5 % compared to the Con-shRNA group on day 5 in U251 (OD value, 1.66 ± 0.04 vs. 2.65 ± 0.08, *p* < 0.001) and U87 (OD value, 0.77 ± 0.03 vs. 2.10 ± 0.08, *p* < 0.001) cells, respectively. Similarly, compared with the Con-shRNA group, the proliferative ability on day 5 in the TMPO-shRNA(S2) group was also observably inhibited by 58.9 % in the U251 cells (OD value, 2.58 ± 0.16 vs. 6.26 ± 0.30, *p* < 0.001, Fig. 3c). Moreover, the colony formation ability of U251 cells infected with the TMPO-shRNA or the TMPO-shRNA(S2) group was further explored, and the size and number of the colony were both smaller in both the TMPO-shRNA and TMPO-shRNA(S2) groups than those in the Con-shRNA group (Fig. 3d). In comparison with the Con-shRNA group, the numbers of colonies were markedly lower in the TMPO-shRNA group (28 ± 6 vs. 128 ± 17, *p* < 0.01) and the TMPO-shRNA(S2) group (4 ± 1 vs. 133 ± 5, *p* < 0.001) in the U251 cells (Fig. 3e). These results suggested that knockdown of TMPO could inhibit the proliferation and colony formation abilities of GBM cells.

### Knockdown of TMPO arrested the cell cycle at the G2/M phase

**Fig. 4:**
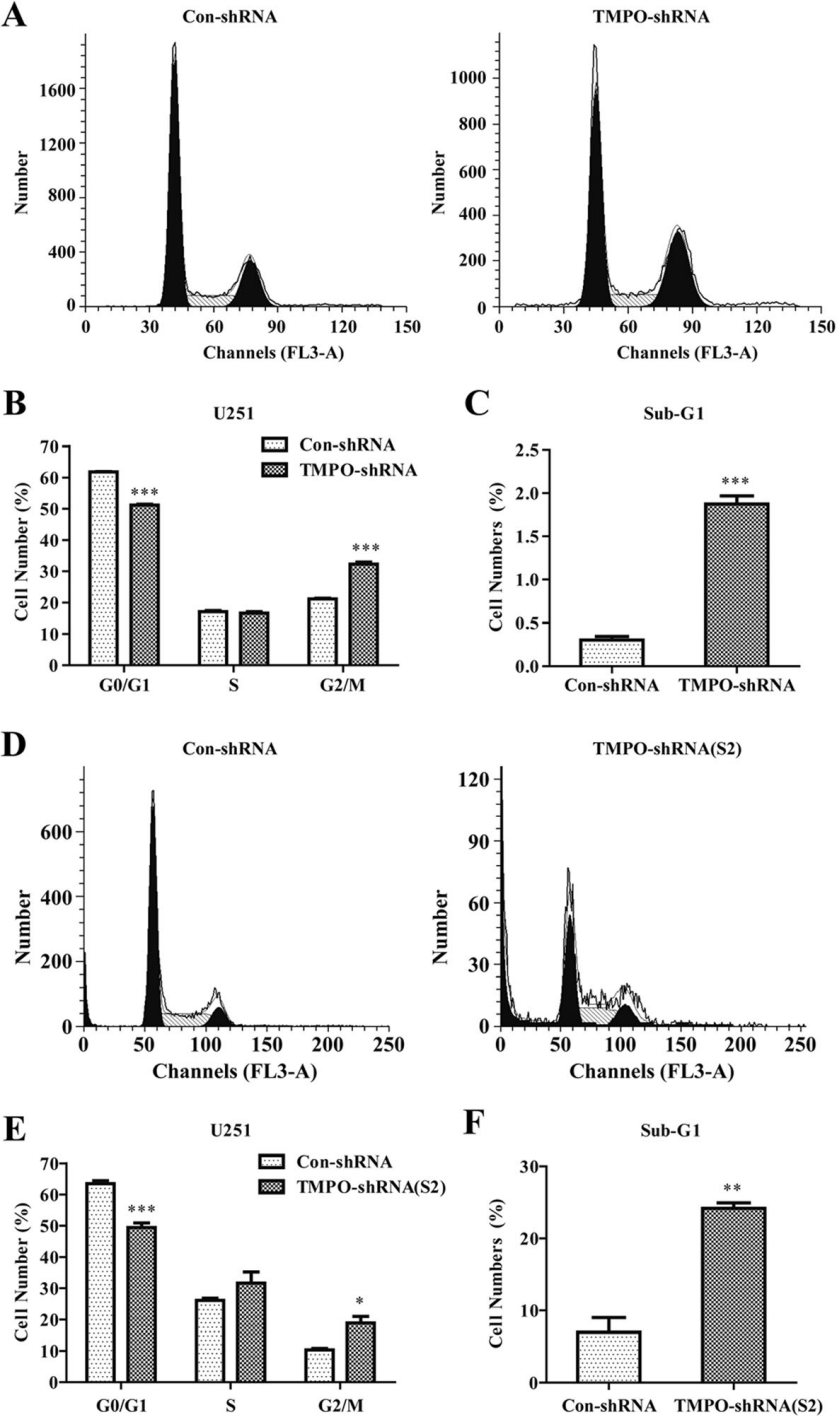
Downregulation of TMPO arrested the cell cycle of U251 cells. **a**, **d** The cell cycle distribution determined by flow cytometer. **b**, **e** Statistical analysis of the cell cycle in the U251 cells with TMPO knockdown. **c**, **f** The respective proportion of sub-G1 phase cells. *p < 0.05, **p < 0.01, ***p < 0.001

The approach of TMPO that regulated cell proliferation was further demonstrated. Cell cycle analysis was performed by flow cytometry in U251 cells after TMPO knockdown (Fig. 4a, d). As shown in Fig. 4b, the knockdown of TMPO in U251 cells decreased the population of the G0/G1 phase (51.2 ± 0.3 % vs. 61.8 ± 0.1 %, *p* < 0.001) and increased the population of the G2/M phase (32.3 ± 0.7 % vs. 21.1 ± 0.3 %, *p* < 0.001) in comparison with the Con-shRNA group. Similar to the results of the TMPO-shRNA group in the U251 cells, a visible decrease in the cell proportion in the G0/G1 phase (49.5 ± 1.4 % vs. 63.5 ± 0.9 %, *p* < 0.001) was observed but an increase in the G2/M phase (18.9 ± 2.1 % vs. 10.4 ± 0.5 %, *p* < 0.05) in the TMPO-shRNA(S2) group compared with the Con-shRNA group (Fig. 4d). But the S phase population did not change in both the TMPO-shRNA and TMPO-shRNA(S2) groups. This suggested the cell cycle was arrested at the G2/M phase by TMPO knockdown. Furthermore, the apoptotic population of U251 cells in the sub-G1 phase was significantly increased in both the TMPO-shRNA (1.9 ± 0.1 % vs. 0.3 ± 0.0 %, *p* < 0.001) and TMPO-shRNA(S2) groups (24.2 ± 0.8 % vs. 7.0 ± 2.0 %, *p* < 0.01) in comparison with the Con-shRNA groups (Fig. 4c, f). These results suggested that TMPO knockdown could inhibit GBM cell growth via inducing cell cycle arrest.

### Knockdown of TMPO promoted cell apoptosis

**Fig. 5:**
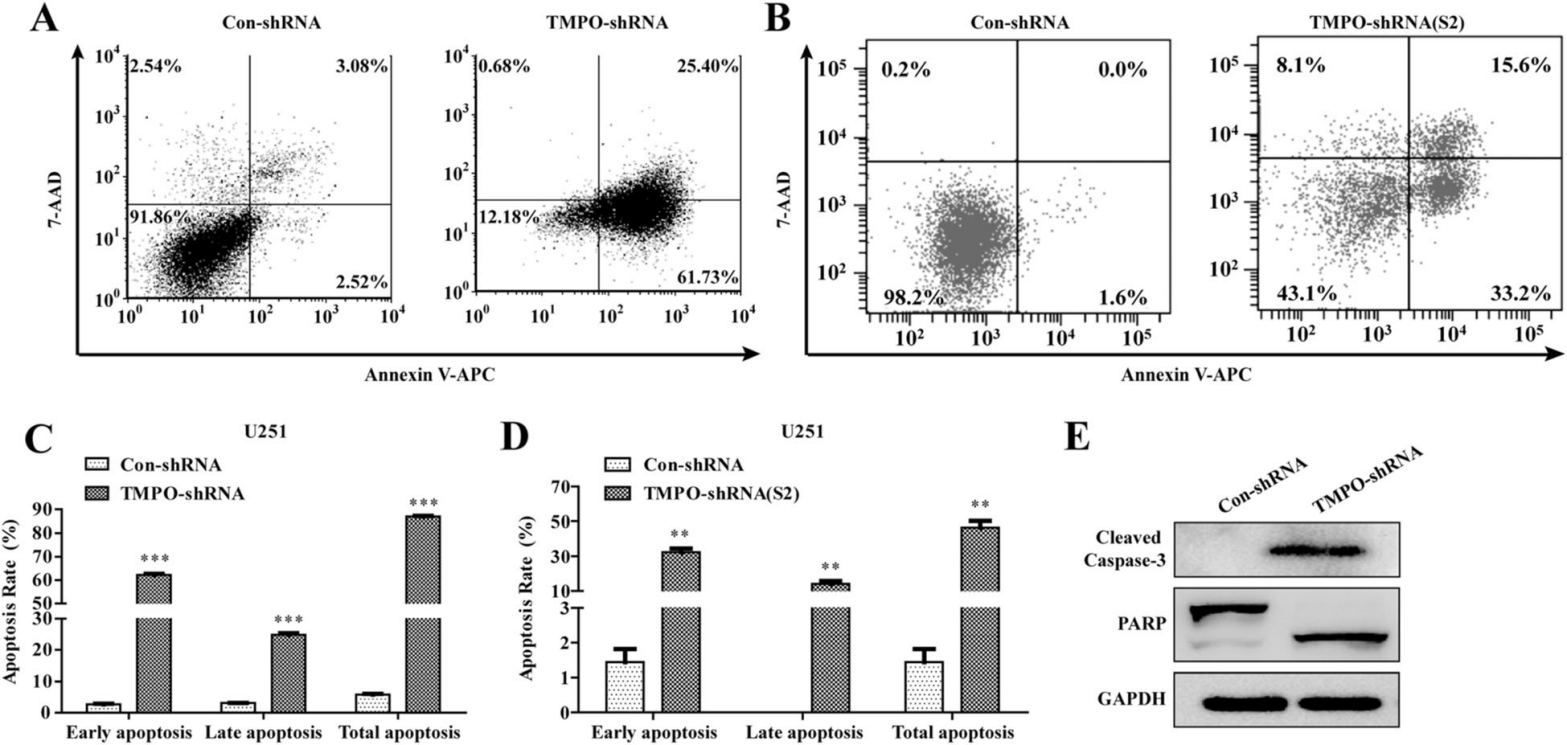
Downregulation of TMPO induced the cell apoptosis of U251 cells. **a**, **b** Representative images showing Annexin V/7-ADD staining results in TMPO-shRNA- or TMPO-shRNA(S2)-infected U251 cells. **c**, **d** Statistical analysis of apoptosis in the U251 cells with TMPO knockdown. **e** The protein expression of cleaved PARP and cleaved caspase-3 in U251 cells was significantly increased after knockdown of the TMPO gene as verified by western blot. **p < 0.01, ***p < 0.001

The effect of TMPO silencing on cell apoptosis was further confirmed using an apoptosis detection kit, and it was found that knockdown of TMPO in U251 cells increased early apoptotic cells (Annexin V^+^/7-AAD^−^) from 2.7 ± 0.2 % to 62.2 ± 0.5 % (*p* < 0.001) and late apoptotic cells (Annexin V^+^/7-AAD^+^) from 3.1 ± 0.0 % to 24.82 ± 0.5 % (*p* < 0.001) and increased nearly 15-fold the total cell apoptotic population of the TMPO-shRNA group compared to the Con-shRNA group (*p* < 0.001) (Fig. 5a, c). Moreover, the cell percentage was more in the early (32.4 ± 2.1 % vs. 1.4 ± 0.4 %, *p* < 0.01) and late (14.1 ± 1.7 % vs. 0.0 ± 0.0 %, *p* < 0.01) apoptosis in the TMPO-shRNA(S2) group than in the Con-shRNA group, and there was an over-32-fold increase in the total cell apoptotic population of the TMPO-shRNA(S2) group compared to the Con-shRNA group (*p* < 0.001) (Fig. 5b, d). The results revealed that TMPO silencing produced a strong cell apoptotic effect in GBM cells.

To explore the role of related cell apoptotic molecules in the knockdown of TMPO-induced cell apoptosis, the effect of TMPO silencing on two classical cell apoptotic molecules caspase-3 and PARP was examined by western blot assay. Caspase-3 is a key executioner of apoptosis and can break many key proteins for apoptosis such as poly(ADP-ribose) polymerase (PARP) [22, 23]. Cleavage of PARP is a marker for cell apoptosis [24]. As shown in Fig. 5e, the expressions of cleaved caspase-3 and cleaved PARP were remarkably increased once TMPO was knocked down. Taken together, the results suggested that TMPO knockdown in U251 cells promoted cell apoptosis due to the upregulation of cleaved caspase-3 and PARP expression.

## Discussion

In the present study, the role of TMPO in GBM was investigated and it was found that TMPO was overexpressed in GBM tissues. By knockdown of the TMPO expression, cell proliferation was suppressed by the MTT and colony formation assays. In addition, when TMPO was downregulated, the percentage of the S phase population did not change, the percentage of G0/G1 was significantly reduced, and the percentage of G2/M was significantly increased, which suggested that the cell cycle was arrested at the G2/M phase after treatment with TMPO-shRNA. This might be because TMPO regulates nuclear assembly in the mitosis; knockdown of TMPO reduced the rate of nuclear assembly [25].

It has been demonstrated that there is a close relation between cell cycle arrest and DNA damage response [26, 27]. Accumulation of the G2/M phase population is prominently related with apoptosis [28]. The finding that knockdown of TMPO induced cell cycle arrest at the G2/M phase prompted us to examine whether apoptosis occurred in U251 cells. As expected, knockdown of TMPO significantly elevated the percentage of cells in early apoptotic and late apoptotic stages. Western blot further suggested knockdown of TMPO increased the cleavage of caspase-3 and PARP expression. Caspase activation plays a critical role in apoptosis and is divided into two types: initiator caspases (caspases-2, 8, 9, 10) and effector caspases (caspases-3, 6, 7). Both the intrinsic pathway and the extrinsic pathway can activate initiator caspases; initiator caspases then cleave and activate effector caspases. Effector caspases subsequently cleave substrate proteins which are essential for cell survival, such as PARP [29, 30].

PARP is proteolysed by caspase-3 into two fragments, a 24-kDa DNA-binding fragment and an 89-kDa catalytic fragment [24]. The cleavage of PARP is critical for apoptosis because it can prevent apoptosis induced by energy depletion and cell survival induced by DNA repair [31, 32]. The results further confirmed that TMPO knockdown promoted apoptosis. The new targets for GBM therapy are critical; inhibition of TMPO could induce apoptosis, suggesting it might be a novel target for GBM treatment.

## Conclusions

This study found TMPO is significantly upregulated in GBM tissues. Functional analysis indicated that knockdown of TMPO suppressed GBM cell proliferation by inducing cell cycle arrest and apoptosis. Although the precise mechanisms are not yet fully understood, this finding suggests that TMPO might play an important role in regulating the proliferation of glioma cells and serve as a new target for GBM treatment.
